# Center Stage: Putting Obesity Staging Systems Into the Spotlight

**DOI:** 10.5888/pcd22.250222

**Published:** 2025-08-28

**Authors:** Sohail Zahid, Allison W. Peng, Alexander C. Razavi, Zhiqi Yao, Roger S. Blumenthal, Michael J. Blaha

**Affiliations:** 1 Ciccarone Center for the Prevention of Cardiovascular Disease, Johns Hopkins University School of Medicine, Baltimore, Maryland; 2 Emory Clinical Cardiovascular Research Institute, Emory University School of Medicine, Atlanta, Georgia

For the past 5 decades, obesity has been primarily defined by body mass index (BMI, calculated as weight in kilograms divided by height in meters squared) (1). In this classification, obesity is categorized as a BMI of 30 or higher, with the following subcategories: class I (BMI of 30–34.9), class II (BMI of 35–39.9), and class III (BMI of ≥40) (2). This classification originated from Metropolitan Life Insurance actuarial tables in the 1950s and was endorsed by the National Institute of Health in the 1980s (3). The World Health Organization (WHO) further promoted this BMI classification in the 1990s, leading to its widespread adoption in biomedical research, clinical practice, and public health policy (1,3). Consequently, the BMI-based classification of obesity became the de facto global standard.

However, BMI has substantial limitations in accurately characterizing obesity at the individual level (4). BMI cannot distinguish between lean muscle and fat, assess body shape, evaluate metabolic capacity, or provide information about adiposity-related organ dysfunction (4). For example, a high-performance athlete may have an elevated BMI, but little body fat (5). In contrast, an older adult with sarcopenic adiposity with low muscle and bone mass may have a “normal” overall weight (6). Certain racial and ethnic populations, such as East or South Asian people, have increased visceral adipose tissue pad deposits at lower BMI levels and have an underestimated level of obesity according to the WHO definition (7). In epidemiological studies, BMI has modest sensitivity with body fat (8) and a U-shaped association with prognosis (9), as people with both low and elevated BMI levels have worse outcomes. These limitations raise concerns about the practical utility of BMI for risk stratification.

To address the concerns of using BMI as a standalone measure of obesity, the Lancet Commission provided a new definition of obesity, stratified by preclinical and clinical obesity, with goals to characterize obesity as a distinct illness and create a diagnostic framework (10). Obesity is defined as excess adiposity assessed either by direct measurement (eg, using a DEXA [dual-energy X-ray absorptiometry] scan) or with at least 2 anthropometric measurements (ie, BMI, waist circumference [WC], waist-to-hip ratio [WHR], or waist-to-height ratio) (10). The Lancet Commission also provided ethnicity-specific recommendations to characterize obesity based on various geographic areas, with a focus on people who exhibit maladaptive consequences of adiposity at lower weight (10). Clinical obesity is present when adiposity results in organ dysfunction or significant physical limitation, while preclinical obesity has no deficits (10). Although these additional components complicate diagnosis, they are essential for accurate characterization of obesity.

We highlight an area for potential improvement using the Lancet Commission definition in classifying illness severity among people with clinical obesity. For example, while heart failure and chronic urinary incontinence both classify people into clinical obesity when due to excess body fat, these conditions have substantially disparate levels of cardiovascular risk and management approaches (11). The Lancet Commission acknowledges this concern, noting that specific treatment recommendations for clinical obesity are outside the scope of the document (10).

One solution involves incorporating a staging system, such as the Edmonton Obesity Staging System (EOSS) (12). EOSS categorizes obesity into 5 stages based on illness severity, incorporating physical limitation, clinical status, and mental health. In EOSS, stage 0 includes no obesity-related health issues, stage 1 includes risk factors, stage 2 includes established comorbidities, stage 3 includes chronic diseases, and stage 4 includes end-stage diseases (12).

The inspiration for EOSS was to follow a framework like those for other chronic diseases that integrates complementary data to assess severity (11). For example, oncology incorporates tumor location, node involvement, and metastasis to grade cancer severity. Obesity is a multifactorial disease that cumulatively causes organ dysfunction, mental illness, and physical disability, so EOSS incorporates these elements to assess overall disease burden (12).

EOSS is a strong predictor of mortality, cardiovascular events, and health care use (13–16) but is not intended to diagnose obesity (11,17). This schema was intended to be a supplement to grade the functional consequences of excess fat mass (11,17), similar to how the New York Heart Association classes categorize the functional symptoms of heart failure and the American Heart Association stages denote disease progression (18).

The Figure shows examples of the Lancet Commission obesity definition with EOSS. A football athlete with a BMI of 32, WC of 32 inches, and WHR of 0.8 with no symptoms of obesity-related conditions was in the WHO class I obesity category but now is classified with no obesity. An older woman with a BMI of 27, WC of 36 inches, and WHR of 1.0 and diabetes was in the WHO overweight category but now has stage 2 clinical obesity. A woman with a BMI of 33, WC of 38 inches, and WHR of 0.9, and prediabetes but no other deficits was in the WHO class I obesity category but now has stage 1 preclinical obesity. Finally, a man with a BMI of 35, WC of 40 inches, and WHR of 1.2, and heart failure was in the WHO class II obesity category but now has stage 3 clinical obesity.

**Figure:**
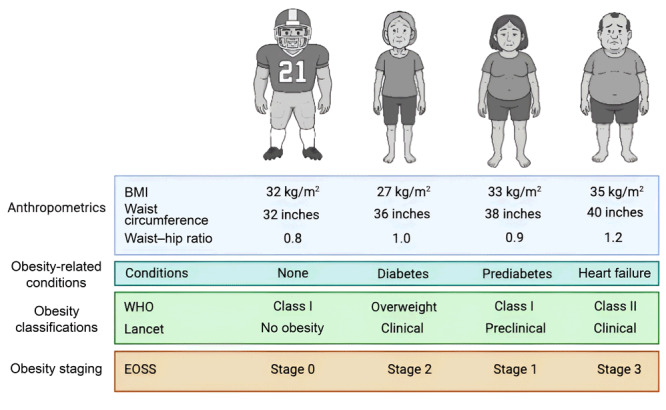
Schematic of obesity definitions and staging across clinical scenarios using guidelines of the Lancet Commission, the World Health Organization, and the Edmonton Obesity Staging System.

A combination of the Lancet Commission definition with an obesity staging system like EOSS can better personalize obesity diagnosis, disease burden, prognosis, and treatment compared with BMI alone (10,11,17). However, debates, challenges, and unanswered questions remain about these new classifications (19,20). Some societies recommend the cardiovascular–kidney–metabolic (CKM) syndrome and systemic metabolic disorder (SMD) schemas, which prioritize assessment of organ systems affected by obesity (13,17). The Lancet Commission and EOSS recommend a holistic assessment of obesity. An ongoing challenge is practical implementation of these definitions into clinical practice and public policy (21). Although BMI is a less accurate surrogate of body fat than direct imaging, it is easy to measure (4), and most people with elevated BMI also have excess adiposity with direct fat measurement. However, recent studies have also shown that excess fat is common at lower weight levels, particularly in people who are older, are of East or South Asian ancestry, or who have been economically or socially marginalized; these populations may have more to gain from improved screening, diagnosis, and treatment (22–24). Identifying the best staging criteria will become even more important to help identify people who might benefit most from novel antiobesity pharmacologic agents and bariatric surgery (11,17). Classifying people into obesity stages such as with EOSS is straightforward at the ends of the schema spectrum, where people exhibit no symptoms or have end-stage disease, but can be challenging in intermediate stages like stages 2 or 3, where the distinction between mild and moderate disease is subtle or subjective. Nonetheless, although these classifications are more time consuming to evaluate, they are major steps forward to prioritize attention on the maladaptive consequences of adiposity and away from a flawed BMI-focused framework.

Although obesity staging systems have been referenced in some clinical guidelines and implemented in select clinical workflows (14,21), their widespread adoption into routine management is limited. We recommend further investigation in clinical and epidemiological studies to assess whether new obesity definitions and staging schema should be in the spotlight of clinical care.

Obesity is an increasingly prevalent chronic disease that is a major contributor to increased rates of illness and death. Despite recognition of the harmful effects of obesity on health outcomes, current public health policies rely on a flawed definition reliant on BMI thresholds. The Lancet Commission issued a new framework for obesity, stratifying people into preclinical and clinical groups, depending on the presence of physical limitation and organ damage. Although this definition more comprehensively defines obesity, staging systems can serve as an adjunct to improve risk stratification and allocate limited clinical resources. We recommend further investigation with updated guidelines, clinical integration, and medical education to assess whether these new obesity definitions and schema can improve our management of this challenging public health epidemic.
